# Microbial metabolite butyrate facilitates M2 macrophage polarization and function

**DOI:** 10.1038/srep24838

**Published:** 2016-04-20

**Authors:** Jian Ji, Dingming Shu, Mingzhu Zheng, Jie Wang, Chenglong Luo, Yan Wang, Fuyou Guo, Xian Zou, Xiaohui Lv, Ying Li, Tianfei Liu, Hao Qu

**Affiliations:** 1 Institute of Animal Science, Guangdong Academy of Agricultural Sciences, 1 Dafeng 1st Street, Wushan, Tianhe District, Guangzhou 510640, China; 2 State Key Laboratory of Livestock and Poultry Breeding, Guangzhou 510640, China; 3 Institute of Immunology, Zhejiang University School of Medicine, Hangzhou, China

## Abstract

Metabolites from intestinal microbes modulate the mucosal immune system by regulating the polarization and expansion of T cells. Whether the microbial metabolites influence macrophage polarization, however, is poorly understood. Here, we show that the large bowel microbial fermentation product, butyrate, facilitates M2 macrophage polarization, *in vitro* and *in vivo*. The supernatant from butyrate-treated M2 macrophage increased the migration and enhanced the wound closure rate of MLE-12 cells. Butyrate attenuated intestinal inflammation in mice with dextran sulfate sodium (DSS)-induced colitis, with a significant increase in colonic expression of the M2 macrophage-associated protein, Arg1. M2 macrophage treated with butyrate, had increased activation of the H3K9/STAT6 signaling pathway, suggesting a mechanism for butyrate facilitated M2 macrophage polarization. Collectively, our study indicated that commensal microbe-derived butyrate is a novel activator of STAT6-mediated transcription through H3K9 acetylation driving M2 macrophage polarization, and delineated new insights into the immune interplay underlying inflammatory bowel disease.

The intestinal immune system associates with the gut microbiota for the maintenance of intestinal health. Imbalance in gut microbiota constituents provokes proinflammatory responses and induces diseases such as inflammatory bowel disease (IBD) in the host^1^. Within the gut microbiota, members of the genus *Clostridia* breakdown indigestible dietary fibers, yielding short-chain fatty acids (SCFAs) as fermentation products, ranging in concentration from 50 to 100 mM in the colonic lumen^2^,^3^. SCFAs have the potential to modulate the mucosal immune system by regulating the differentiation and expansion of T cells. In mice, SCFAs regulate the size and function of the colonic regulatory T-cell (Treg) pool and protect against colitis in an *Ffar2*-dependent manner. The large bowel microbial fermentation product, butyrate induces functional colonic Treg via T-cell intrinsic epigenetic up-regulation of the *Foxp3* gene^4^,^5^,^6^.

It remains poorly understood, however, whether such metabolites, including butyrate, influence macrophage polarization. Macrophages are key components of the innate immune system and provide protection against a wide variety of infections and critically shape the inflammatory environment in many tissues. Depending on the microenvironment, macrophages have been classified as classically activated M1 or alternatively activated M2. M1 macrophages are induced by inflammatory stimuli, such as lipopolysaccharide (LPS), and are characterized by the production of high levels of proinflammatory cytokines, including, interleukin (IL)-1β, IL-12, and tumor necrosis factor (TNF)-α, inducible nitric oxide synthase, (iNOS), reactive oxygen species (ROS), and suppression tumor cell growth^7^,^8^. The M2 phenotype is induced by the Th2 cytokines, IL-4 or IL-13, and is characterized by production of arginase 1 (Arg1) and found in inflammatory zone 1 (Fizz1), which are important for the resolution of inflammation, wound healing, and tissue repair^9^,^10^,^11^. Previous studies have demonstrated that butyrate had anti-inflammatory effects on LPS-mediated M1 macrophage via reducing production of proinflammatory mediators such as NO and IL-6^12^,^13^. However, there is limited information about the effect of butyrate on M2 macrophage polarization and its function. Here, we provide evidence that commensal microbe-derived butyrate facilitates the polarization of M2 macrophage, which plays a critical role in dextran sulfate sodium (DSS)-induced colitis.

## Results

### Butyrate facilitates polarization of M2-BMDMs

**Figure 1:**
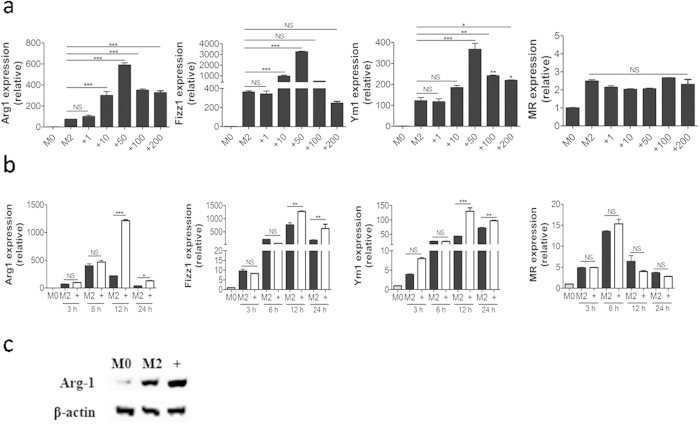
Butyrate increases the expression of genes typical of M2 macrophage. (**a**) M0-BMDMs were directly exposed in series concentrations of butyrate for 24 h. Expression of *Arg1, Fizz1, Ym1*, and *MR* was measured by quantitative RT-PCR. (**b**) The relative quantity of *Arg1, Fizz1, Ym1*, and *MR* mRNA transcribed by nonpolarized (M0-BMDMs, gray bars) or M2-BMDM after 3, 6, 12, and 24 h with IL-4 in the absence (white bars) or presence (black bars) of 50 μg/mL butyrate. (**c**) Arg1 protein levels in the absence or presence of 50 μg/mL butyrate in M2-BMDMs after 24 h treatment. Data are mean ± SD for at least three independent experiments. Comparisons between means used *t* tests (*P < 0.05, **P < 0.01, ***P < 0.001).

The cells were morphologically typical of bone marrow-derived macrophages (BMDMs) (Supplementary Fig. 1a) and 99.9% were F4/80^+^ as determined by flow cytometry (Supplementary Fig. 1b,c). Flow cytometry and Cell Counting Kit-8 assays showed no changes in BMDMs’ viability following butyrate treatment (Supplementary Fig. 2). To investigate the effect of butyrate on polarization of M2 macrophage, we first examined the hallmarks of M2 macrophage expression in BMDMs directly treated with butyrate. The results showed that treatment of M0 macrophages with butyrate did not stimulate the upregulation of M2 markers (*Arg1, Fizz1, Ym1* and *MR*) (Supplementary Fig. 3). The data suggested that butyrate did not itself drive polarization of M0 macrophages towards the M2 phenotype. To determine whether butyrate facilitated IL-4–driven M2 polarization, M0 macrophages were activated by IL-4 in the presence of various concentrations (1 to 200 μg/ml) of butyrate. The relative expression of genes encoding *Arg1, Fizz1*, and *Ym1,* but not *MR*, was considerably higher in M2-BMDMs exposed to 50 μg/ml butyrate for 24 h (Fig. 1a). Meanwhile, stimulation with butyrate did not alter the surface expression of costimulatory molecules, CD80 or CD86, on M2-BMDMs (Supplementary Fig. 4). Butyrate clearly increased the expression of M2 macrophage marker genes in M2-BMDMs and 50 μg/mL was then used to determine its influence the progress of polarization, again triggered by IL-4. Compared to its absence, exposure of M2-BMDMs to butyrate significantly enhanced expression of *Arg1, Fizz1*, and *Ym1* after 12 h treatment with IL-4 and the effect on *Arg1* was most striking (Fig. 1b). Western blot analysis of Arg1 protein content at 24 h was consistent with the enhanced gene expression (Fig. 1c). Collectively, these data indicated that butyrate enhanced the IL-4-induced expression of M2-associated markers.

### Butyrate enhances migration and wound-healing properties in M2-BMDMs

**Figure 2:**
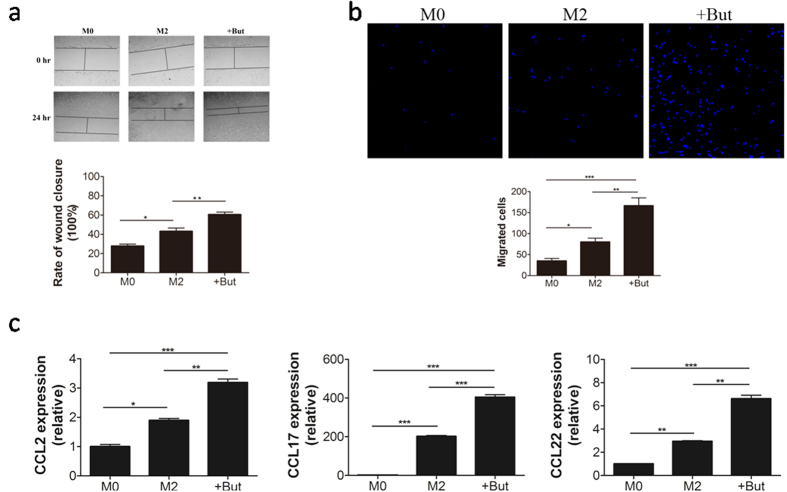
Wound healing and cell migration in MLE-12 cell cultures. (**a**) Wounded MLE-12 cells were treated with supernatants conditioned for 24 h by non-polarized (M0) or M2-BMDMs cells in the absence (M2) or presence (+But) of butyrate. Photographs (×100) were taken at 0 and 12 h, after the wound was made. The extent of wound closure of MLE-12 was measured and expressed as a percentage. (**b**) Transwell filter assay was used to measure chemoattraction and migration of MLE-12 cells to lower wells containing conditioned media from non-polarized (M0) or M2-BMDM cells in the absence (M2) or presence (+But) of butyrate. Migrated cells at the lower surface of the transwell filter were stained with DAPI and counted. (**c**) Total RNA was isolated from M0-BMDMs, and M2-BMDMs, untreated or treated with butyrate, and analyzed by RT-PCR for the expression of representative M2 macrophage chemokine genes. Data are shown as mean ± SD for three independent experiments, Comparisons between means used *t* tests (*P < 0.05, **P < 0.01, ***P < 0.001).

M2 macrophage promotes the resolution of wound healing by facillating cell migration to damaged tissues^14^,^15^. To analyze if butyrate influences M2 macrophage function, wound-healing assay was performed using MLE-12 bronchial cells in an *in vitro* scrape model. As shown in Fig. 2a, supernatants from M2-BMDMs increased wound closure compared with those from the M0-BMDMs (*p* < 0.05) and this effect on re-epithelialization was improved further when M2-BMDMs had been treated with butyrate. As shown in Fig. 2b, medium conditioned by M2-BMDMs for 24 h promoted the migration of MLE-12 cells in transwell filter assays. The number of MLE-12 cells in the permeating septum after 12 h more than doubled (*p* < 0.05) in the presence of medium conditioned by M2-BMDMs rather than M0 and doubled again (*p* < 0.01) when conditioning had occurred in the presence of butyrate. Together, these data demonstrated that butyrate significantly enhanced the functional ability of M2-BMDMs promote cell migration and wound-healing. Consistent with this, the expression of relevant C-C cytokine genes, *CCL2, CCL17*, and *CCL22* was consistently higher in butyrate-treated M2-BMDMs (Fig. 2c).

### Butyrate ameliorates dextran sulfate sodium (DSS)-induced colitis in mice

**Figure 3:**
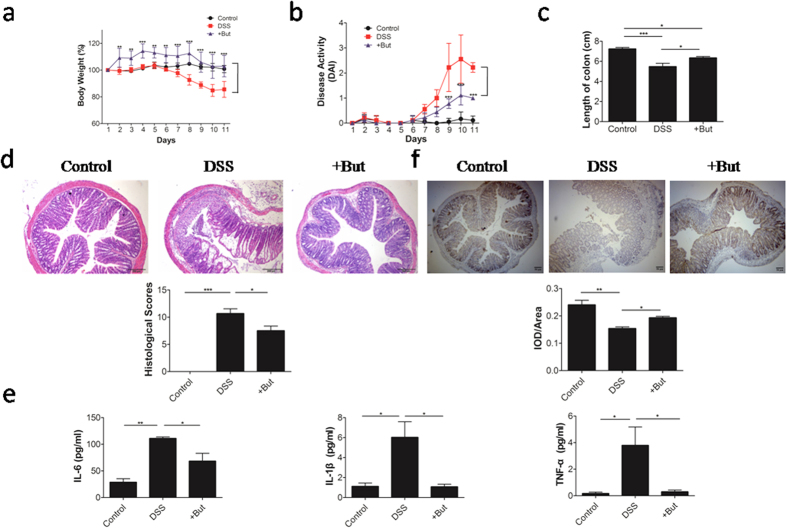
Effect of butyrate on dextran sulfate sodium (DSS)-induced colitis. (**a**) Relative body weight change, (**b**) disease activity index, (**c**) and colon length of mice with colitis treated with butyrate (+But) or left untreated (DSS), and healthy control mice (control). (**d**) Histological appearance and histological scores of colons from control mice, and mice with colitis, either treated with butyrate or untreated. (**e**) IL-1β, IL-6, and TNF-α in the serum of mice with colitis, treated with butyrate or untreated, and control mice. (**f**) Quantitation of the colonic Arg1 protein expression in serial sections by immunohistochemistry. The mean integrated optical density (IOD) (IOD/total area) represents the Arg1 expression level. Data are representative of 6 mice/group, with similar staining. Data are shown as mean ± SD for three independent experiments, Comparisons between means used *t* tests (*P < 0.05, **P < 0.01, ***P < 0.001).

M2 macrophage plays an important role in colonic infection with parasitic nematodes^16^. Furthermore, an increase in intestinal M2 macrophage has been associated with reducing the severity of colitis in mice^17^. We first evaluated the therapeutic effect of butyrate on DSS-induced colitis as a model of inflammatory bowel disease (IBD). Butyrate-treated mice showed much milder symptoms than the untreated colitis control mice, as evidenced by a slower rate of weight loss (Fig. 3a) and a lower disease activity index (DAI; Fig. 3b). Meanwhile, butyrate could promote the growth in mice and the results are consistent with the butyrate can improve the growth performance of animals^18^,^19^,^20^. Histological examination of the colons showed less mucosal damage, demonstrating the protective effect of butyrate against colitis in the DSS-induced colitis model (Fig. 3c,d). Following butyrate treatment, reduced levels of proinflammatory cytokines, namely IL-6, TNF-α, and IL-1β, were detected in the serum compared with control mice (Fig. 3e). Moreover, we found that butyrate-treated mice had more Arg1 protein expression in colonic tissues (Fig. 3f). Taken together, these data showed that butyrate-treated mice have reduced clinical disease and histological damage compared with control mice, and this protection during DSS-induced colitis may correlate with increased in Arg1 protein expression in the colon during inflammation.

### M2 macrophage confers protection to mice during DSS-induced colitis

**Figure 4:**
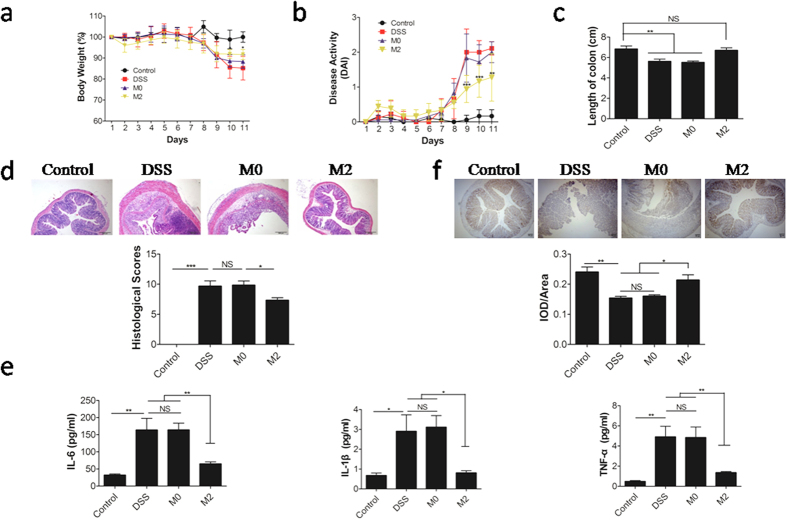
Therapeutic effect of M2-BMDMs on DSS colitis. DSS was administered to mice to induce colitis and M0 or M2-BMDMs were transferred intravenously. (**a**) Relative body weight change, (**b**) disease activity index, and (**c**) colon length of mice with colitis, treated with M0 or M2 BMDMs or untreated (DSS), and control mice. (**d**) Histological appearance and histological scores of colons. (**e**) IL-1β, IL-6, and TNF-α in serum. (**f**) Quantitation of Arg1 protein expression in serial sections by immunohistochemistry. The mean IOD (IOD/total area) represents the Arg1 expression level. Data are representative of 6 mice/group, with similar staining. Data are shown as mean ± SD for three independent experiments, Comparisons between means used *t* tests (*P < 0.05, **P < 0.01, ***P < 0.001).

To further elucidate the importance of M2-BMDMs in IBD, we adoptively transferred M0- and M2-BMDMs into colitis-bearing recipient mice. There was no significant difference between the mice treated with M0-BMDMs and the colitis-only control group. In contrast, mice receiving M2-BMDMs showed significantly less weight loss compared with the colitis-only control group, with lowered DAI (Fig. 4a,b) and improved histological condition of the colon (Fig. 3c,d). Moreover, the M2-BMDM recipient mice possessed less IL-6, TNF-α, and IL-1β in the serum than the colitis-only control mice (Fig. 4e). Furthermore, increased Arg1 protein expression was detected in the colonic tissues of M2-BMDM recipient mice than in control tissues (Fig. 4f). These data demonstrated that M2 macrophage can protect against DSS-induced colitis in mice.

### Regulation of STAT6 by butyrate in M2-BMDMs

**Figure 5:**
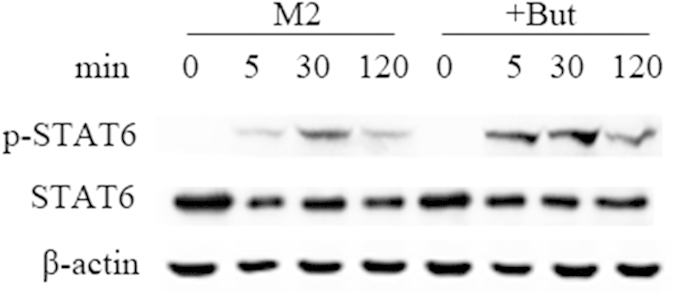
Butyrate promotes IL-4-induced STAT6 signaling pathways in M2 macrophage. M2-BMDMs were incubated with IL-4 and IL-4 with butyrate for the indicated time periods. Cell lysates were prepared and blotted with anti-phospho-STAT6. Total STAT6 and β-actin were probed as quantitative controls. Data are representative of three independent experiments.

STAT6 is a critical transcription factor for the IL-4-mediated signaling response in M2 macrophage polarization^21^, therefore, we compared phosphorylation of STAT6 in butyrate-treated M2-BMDMs and untreated M2-BMDMs. We observed an increase in STAT6 phosphorylation in response to IL-4 in butyrate-treated M2-BMDMs compared with M2-BMDMs (Fig. 5). Therefore, the higher expression of M2 macrophage marker mRNA may be a consequence of increased STAT6 phosphorylation.

### Butyrate reduces histone H3K9 deacetylase in M2-BMDMs

**Figure 6:**
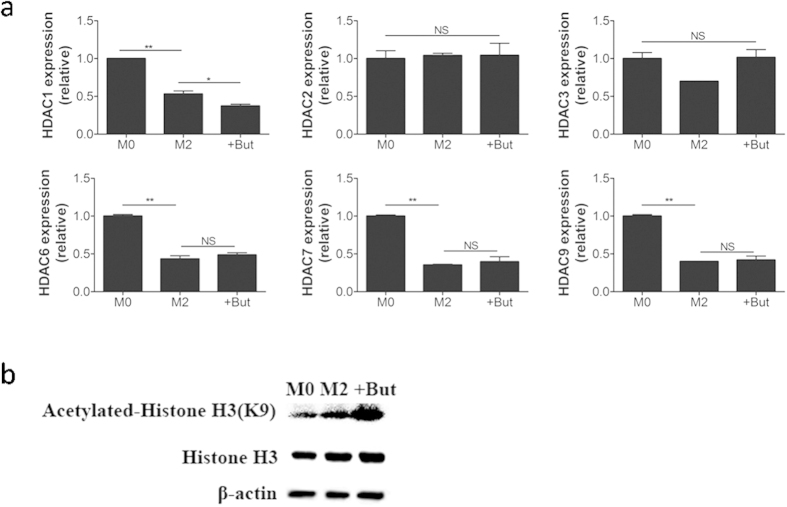
Butyrate increases acetylation of H3K9. (**a**) M2-BMDMs cultured in the presence of butyrate for 6 h were examined for expression of *HDAC 1, 2, 3, 6, 7*, and *9* by quantitative RT-PCR. Data are the mean ± SD of three independent experiments. (**b**) M2-BMDMs were treated with butyrate for 12 h. The level of H3K9 acetylation were assessed by immunoblot. Total histone and β-actin levels were used as loading controls. Data are representative of three independent experiments.

Butyrate, as a histone deacetylase (HDAC) inhibitor, is known to regulate gene expression epigenetically^22^,^23^. Whether butyrate alters the histone acetylation of BMDMs to epigenetically regulate the transcription of genes responsible for M2-BMDM development or function is still unknown. We hypothesized that butyrate enhanced the polarization of M2 macrophage involved the HDACs inhibition. To test this hypothesis, M2-BMDMs were stimulated with butyrate for 6 h and the expression of *HDAC* genes was analyzed. The expression of *HDAC1, HDAC6, HDAC7*, and *HDAC9* was reduced in M2-BMDMs, while no differences were observed in *HDAC2* and *HDAC3* gene expression. Butyrate-treated M2-BMDMs had lower *HDAC1* gene expression than M2-BMDMs (Fig. 6a). Western blot analysis demonstrated that butyrate treatment of M2-BMDMs enhanced histone 3 lysine 9 acetylation (H3K9) (Fig. 6b). These results suggested that butyrate enhanced M2-BMDMs polarization partly through *HDAC1* inhibition and histone H3K9 acetylation.

### Histone H3K9 acetylation is required for M2 macrophage polarization

**Figure 7:**
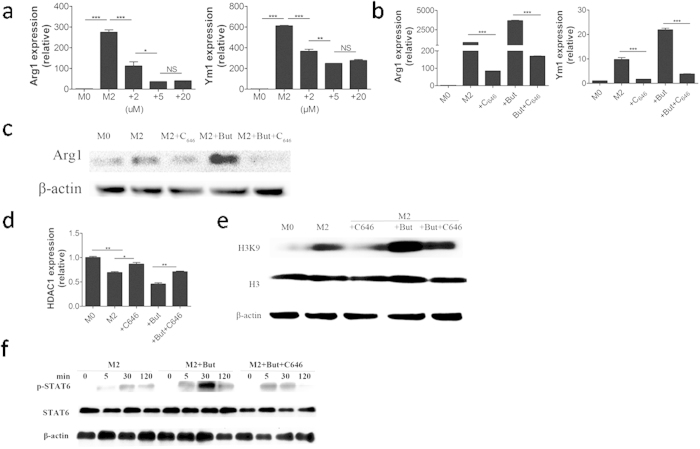
Histone acetyltransferase (HAT) inhibition mediates butyrate-treated M2 macrophage polarization by inhibiting the expression of acetylated H3K9. (**a**) *Arg1* and *Ym1* gene expression in butyrate-treated M2 macrophage treated with increasing concentrations (2–20 μM) of HAT inhibitor, C646. (**b**) *Arg1* and *Ym1* gene expression in the presence of butyrate (50 μg/ml) and C646 (5 μM). (**c**) Western blot analysis of Arg1 protein in butyrate-treated M2-BMDMs after 12 h treatment with C646 (5 μM). (**d**) Quantitative RT-PCR analysis of *HDAC1* gene expression in butyrate-treated M2-BMDMs after 6 h treatment with C646 (5 μM). (**e**) Western blot analysis of acetylated H3K9, total histone H3, and β-actin proteins in butyrate-treated M2-BMDMs after 12 h treatment with C646 (5 μM). Cells were lysed and western blotting performed with the indicated antibodies. (**f**) Phosphorylation of STAT6 in butyrate-treated M2-BMDMs. Western blotting was performed with anti-phospho-STAT6, STAT6, and β-actin. Data are representative of three independent experiments.

To investigate whether acetylation of H3K9 was involved in M2 macrophage polarization, the p300/CBP specific histone acetyltransferase (HAT) inhibitor, C646, was used to reduce the acetylation of H3K9. We observed that with increasing concentrations (2–20 μM) of C646, *Arg1* and *Ym1* mRNA expression was decreased (Fig. 7a). Additionally, C646 inhibited the polarization of M2-BMDMs and M2-BMDMs treated with butyrate (Fig. 7b). Additionally, the Arg1 protein level was dramatically decreased in butyrate-treated M2-BMDMs with concomitant C646 treatment, compared with untreated M2-BMDM controls (Fig. 7c). Meanwhile, C646 effectively enhanced the expression of *HDAC1* and reduced the acetylation of H3K9 in both M2-BMDMs and butyrate-treated M2-BMDMs (Fig. 7d,e). Moreover, we found lower STAT6 phosphorylation in response to C646 in butyrate-treated M2-BMDMs compared with untreated M2-BMDMs. Together, we concluded that butyrate mediated its effects on M2 macrophage largely through its HDAC inhibitor activity augmenting M2 macrophage polarization by the STAT6 signaling pathway.

## Discussion

In this study, we have identified that a metabolite derived from commensal microbes, butyrate, effectively facilitates M2 macrophage polarization. Although butyrate did not directly affect the polarization of M0-BMDMs to M2-BMDMs, it could promote a greater degree of polarization in IL-4-induced M2 macrophage. Additionally, butyrate enhanced the critical functions of M2 macrophage by improving re-epithelialization and the migration of MLE-12 cells. Furthermore, we demonstrated that butyrate attenuates intestinal inflammation in mice DSS-induced colitis model partly via M2 polarized macrophage.

The DSS-induced colitis model is suitable to study the contribution of innate immune cells to colitis, particularly to epithelial repair mechanisms^24^,^25^. After DSS-induced gut injury, macrophages are recruited to the colon and play a central role in disease pathogenesis^26^,^27^. Previous studies showed that a defect in M2 macrophage polarization led to increased severity in DSS-induced colitis, whereas adoptive transfer of *in vitro*-derived M2 macrophage could attenuate colonic inflammation in mice^17^,^28^,^29^. Recent evidence showed that another SCFA, acetate, in the drinking water markedly attenuated disease indices, with increased colon length, decreased DAI, and reduced TNF-α levels in germ-free mice treated with DSS^30^. Here, we employed the DSS-induced colitis model and demonstrated that M2 macrophage were indeed responsible for the therapeutic effects of butyrate on IBD. These findings supported the role of M2 macrophage polarization in the development of inflammatory diseases, and they showed that commensal microbe-derived butyrate, as a new effector molecule, could ameliorate disease severity in DSS-induced colitis by enhancing the polarization of M2 macrophage. It has been suggested that Arg1, expressed by M2 macrophage, is important for tissue repair in diverse models and tissues^14^,^15^,^31^,^32^,^33^. Our results show increased Arg1 protein in butyrate-treated mice, associated with less mucosal damage and lower levels of proinflammatory cytokines.

It is well known that the classical pathway for IL-4-induced M2 macrophage polarization involves the STAT6 signaling pathway. The polarization of M2 macrophage is mediated by IL-4-dependent activation of STAT6, which leads to the expression of *Fizz1, Ym-1*, and *Arg1* in macrophages^34^. These results provided a molecular mechanism for the butyrate-mediated protection against DSS-induced colitis, possibly by enhancing STAT6 signaling in intestinal tissues. Another possible mechanism of butyrate-enhanced M2 polarization may be by inhibiting the activity of HDACs. Previous studies have demonstrated that butyrate inhibited the activity of HDACs in many different cell types^22^. H3K9 acetylation is considered a hallmark of transcriptional activation. Another SCFA propionate treatment of *Ffar2*^+/+^ mice reduced Treg *HDAC6* and *HDAC9* expression and enhanced H3K9 acetylation^6^. Treatment of BMDMs with butyrate led to a downregulation of LPS-induced proinflammatory mediators by inhibition of H3K9 acetylation^12^. To determine whether butyrate behaved as an *HDAC* inhibitor involved in the polarization of M2-BMDM, we checked the levels of *HDACs* in M2-BMDMs without butyrate. Compared with M0-BMDMs, the expression of *HDAC1, HDAC6*, and *HDAC9* were decreased. Western blot analysis also demonstrated that H3K9 acetylation was enhanced in M2-BMDMs. Furthermore, *HDAC1* expression was further reduced and the H3K9 acetylation was increased in butyrate-treated M2-BMDMs. The level of histone acetylation is tightly regulated by the opposing activities of HAT and HDACs^35^. C646 is capable of attenuating HDACs, such as in butyrate, trichostatin, and cAMP-induced H3K9 acetylation^36^,^37^. To further determine whether acetylation of H3K9 mediated the polarization of M2 macrophage, we treated M2-BMDMs with C646. We showed that the expression of Arg1 and H3K9 acetylation was decreased in C646-treated M2-BMDMs. Moreover, C646 efficiently inhibited the effect of butyrate on facilitating M2-BMDMs polarization. These results suggest that butyrate may facilitate M2 macrophage partly through *HDAC1* inhibition.

We next investigated how butyrate modulates STAT6 phosphorylation, which is required for IL-4 induced M2-BMDMs polarization. We hypothesized that H3K9 may play an important role in regulating the level of phosphorylated STAT6 and controlling M2 macrophage polarization. Our results showed that C646 could effectively reduce the level of phosphorylated STAT6 in both M2-BMDMs and butyrate-treated-M2-BMDMs. These data demonstrated that butyrate enhanced the STAT6 phosphorylation partly via inhibiting *HDAC1* gene expression and increasing H3K9 acetylation. The STAT6 signaling pathway requires various co-activators or co-repressors to promote and regulate transcription of endogenous genes. Consistent with this concept, The polypyrimidine tract binding protein (PTB)-associated splicing factor (PSF) recruits *HDAC1* to the STAT6 complex, which reduces H3 acetylation at the promoter regions of the Igε gene and inhibits STAT6 signing in lymphomas^38^. IL-4 induced expression of the reticulocyte-type 15-lipoxygenase-1 (*15-LOX-1*) gene requires the enhancement of H3 acetylation and the activation of the STAT6 signaling pathway in A549 lung epithelial cells^39^. Our findings also support the concept that histone acetylation correlates with their post-translational modifications and coordinately regulate STAT6-mediated gene transcription. Butyrate-mediated activation of H3K9 acetylation and the STAT6 signaling pathway, and its influence on the polarization of M2 macrophage are still unclear. SCFAs bind the G-protein-coupled receptor 43 (GPR43; also known as FFAR2), which is expressed on immune cells, mediating resolution of inflammatory responses^6^,^30^,^40^. GPR43 is expressed on the colonic epithelium and butyrate provides a major source of energy for colonocytes^41^. *Gpr43*^*−/−*^ mice showed aggravated or unresolving inflammation in DSS-induced colitis. In a T-cell-dependent model of colitis, *Gpr43*^*−/−*^ mice also had more severe disease with increased colon histological scores^30^. SCFAs mediate the size and function of the colonic Treg pool and protect against colitis in a *Gpr43*-dependent manner in mice^5^,^6^. Therefore, using *Gpr43*^*−/−*^ mice might enable the dissection of a precise mechanism of action for butyrate on H3K9 acetylation/STAT6 signaling in the polarization of M2-BMDMs. Moreover, the inflammatory process is highly complicated, and considerably more information is required for an improved understanding about macrophages *in vivo*. Because macrophages are able to perform mixed M1/M2 phenotypes in pathological conditions, further studies may focus not only on M2 polarization but also on the changes in M1/M2 polarization treated with butyrate during the development of inflammatory bowel disease. In addition, the polarization of either M2 macrophage or Treg could be facilitated or induced by butyrate. Future studies may delineate a dynamic view of this inflammatory process, take M2 macrophage and Treg in systemic and local milieu into account, and define the kinetics, plasticity, reversibility, and memory of their responses in order to full elucidate the mechanism that butyrate ameliorates inflammatory bowel disease in mice.

In sum, our studies indicated that commensal microbe-derived butyrate enhanced the IL-4-mediated STAT6 transcription, achieved through H3K9 acetylation to enhance M2-BMDMs polarization, and delineated new insights into the immune interplay underlying inflammatory bowel disease.

## Methods

### Animals

Female C57BL/6 mice, 8–10 weeks of age, were purchased from Shanghai Laboratory Animals Co., Ltd (SLAC; Shanghai, China). All mice were maintained in the Experimental Animal Center of Zhejiang University. Experiments were conducted in accordance with the Chinese guidelines for animal welfare, approved by the Animal Welfare Committee of Animal Science College, Zhejiang University.

### M2 macrophage culture

Bone marrow-derived macrophages (BMDMs) were generated by isolating bone marrow from femurs and culturing in high glucose Dulbecco’s modified Eagle’s medium (DMEM, Hyclone, Logan, UT, USA) containing 10% (vol/vol) fetal bovine serum (Gibco) with macrophage colony-stimulating factor (M-CSF; 15 ng/mL; Peprotech) at 37 °C with 5% CO_2_.Half of the medium was replaced with fresh medium containing M-CSF every 3 days. After 6 days of culture, IL-4 (15 ng/mL) was added to generate full alternatively activated M2-BMDMs for 24 h. Untreated BMDMs were retained as nonpolarized macrophages (M0-BMDMs). The BMDMs were subsequently cultured for 1.5-24 h in the presence of sodium butyrate (Sigma-Aldrich) or histone acetyltransferase (HAT) inhibitor C646 (Selleck).

### Cell viability assays

Cells (5, 000 cells/well) were seeded into a 96-well plate and treated with variable concentrations of butyrate for 24 h. Subsequently, the cells were labeled using a Cell Counting Kit-8 (CKK8) (Dojindo, Tabaru, Japan) for 2 h. Absorbance at 450 nm was measured with a plate reader and cell viability was expressed as the percentage of the absorbance of treated cells versus untreated cells.

### cDNA synthesis and real-time RT-PCR

Total cellular RNA was extracted with the TRIzolreagent (Invitrogen, USA), and cDNA was synthesized by using M-MLV reverse transcriptase (Takara, Japan). Real-time (RT)-PCR was performed with SYBR Green master mix (Applied Biosystems). To normalize the amount of total RNA in each reaction, *β-actin* cDNA was used as an internal control. Results are expressed as relative abundance, being log (2-ΔΔCt). The RT-PCR primers were synthesized as follows: *β-actin* forward 5′-GATGAGATTGGCATGGCTTT-3′, reverse 5′-CACCTTCACCGTTCCAGTTT-3′; *Arg1* forward 5′-CTGCAGCACTTGGATCAGGAACCTG-3′, reverse 5′-GGAGTAGCCTGTGTGCACCTGGAA-3′; *Ym1* forward 5′-GGATGGCTACACTGGAGAAA-3′, reverse 5′-AGAAGGGTCACTCAGGATAA-3′; *MR* forward 5′-GCAGACTGCACCTCTGCCGG-3′, reverse 5′-TGCTGCTTGCAGCTTGCCCT-3′; *Fizz1* forward 5′-CCCTCCACTGTAACGAAG-3′, reverse 5′-GTGGTCCAGTCAACGAGTAA-3′; *HDAC1* forward 5′-CCAAGTACCACAGCGATGAC-3′, reverse 5′-TGGACAGTCCTCACCACG-3′; *HDAC2* forward 5′-TGAAGGAGAAGGAGGTCGAA-3′, reverse 5′-GGATTTATCTTCTTCCTTAACGTCTG-3′; *HDAC3* forward 5′-CACCATGCCAAGAAGTTTGA-3′, reverse 5′-CCCGAGGGTGGTACTTGAG-3′; *HDAC6* forward 5′-CTGCATGGCATCGCTGGTA-3′, reverse 5′-GCATCAAAGCCAGTGAGATC-3′; *HDAC7* forward 5′-CTCGGCTGAGGACCTAGAGA-3′, reverse 5′-CAGAGAAATGGAGCCTCTGC-3′; *HDAC9*, forward 5′-GCGGTCCAGGTTAAAACAGAA-3′, reverse 5′-GCCACCTCAAACACTCGCTT-3′; *CCL2* forward 5′-GTTGGCTCAGCCAGATGCA-3′, reverse 5′-AGCCTACTCATTGGGATCATCTTG-3′; *CCL17* forward 5′-ATGAGGTCACTTCAGATGCT-3′, reverse 5′-CCTCTGGATAGCGAGGACTG-3′; *CCL22* forward 5′-CATCATGGCTACCCTGCGTGTCCC-3′, reverse 5′-CCTCCTCCCTAGGACAG TTT ATGGA-3′.

### Antibodies

Phospho-STAT6 (Tyr641) and STAT6 antibodies were from Abcam. Antibodies against β-actin were from Santa Cruz Biotechnology, *Histone* H3 lysine 9 acetylation (H3K9) was from Cell Signaling Technologies, and anti-Arg1 was from Signalway Antibody.

### Western blotting

Cell lysates containing extracted proteins were separated on 10% SDS-polyacrylamide gels and transferred to nitrocellulose membranes. Membranes were blocked at room temperature for 1 h in 5% fat-free milk, incubated overnight at 4 °C with a primary antibody, followed by incubation with the appropriate HRP-conjugated reporter antibody for 1 h at room temperature. The enhanced chemiluminescence Fluorchem E system (Cell Biosciences, Santa Clara, CA) was used for developing.

### Flow cytometric analysis

BMDMs were trypsinized and incubated with phycoerythrin-labeled antibodies against mouse F4/80, CD80 and CD86 for 30 min on ice, washed with PBS, and analyzed using a FACSCalibur flow cytometer (Becton Dickinson, Palo Alto, CA) and Cell Quest software (Becton Dickinson).

### Wound-healing assay

The wound-healing assay was performed as previously described *in vitro*^42^. Transformed mouse lung epithelial cells (MLE-12) were grown to confluency in a 24-well plate. Confluent cell monolayers were mechanically wounded by scraping a sterile conventional pipette tip across the monolayer. Wounded MLE-12 monolayers were washed three times with PBS (pH 7.4) to remove loose cells and debris, and then were incubated for 12 h at 37 °C with 5% CO_2_ in the presence of supernatants collected from M2-BMDMs untreated or stimulated with butyrate for 24 h. A magnification of 10× was used to allow a major surface area to be viewed. Photographs were taken and analyzed using Image J software to measure the re-epithelialization area.

### Migration assays

A migration assay of MLE-12 cells was performed using matrix-coated polycarbonate filters (8-μm pore size, 24 well, Transwell; Becton Dickinson). MLE-12 cells were resuspended in 500 μL DMEM without fetal bovine serum and seeded to the upper chamber. Supernatant (500 μL) from M2-BMDMs cultured in the presence or absence of butyrate was added to the lower chamber. After 24 h, cells that had migrated into the lower wells were collected, stained with DAPI (Roche Diagnostics), and counted under a microscope.

### Induction and assessment of DSS-induced colitis

C57BL/6 mice were randomized into groups of three, with a comparable average body weight in each group. Acute colitis was induced by administering 2% wt/v DSS (MW 36 000–50 000; MP Biomedicals, LLC, Eschwege, Germany) in the drinking water for 11 days^43^. Mice received sodium butyrate (150 mM) in the drinking water beginning at day 0.Body weight, rectal bleeding, stool consistency, and survival were monitored daily after DSS administration. The disease activity index (DAI) used to determine clinical score was calculated by grading on a scale of 0–4 for the following variables: change in weight (0, <1%; 1, 1–5%; 2, 5–10%; 3, 10–20%; and 4, >20%), intestinal bleeding (0, negative; 4, positive), and stool consistency (0, normal; 2, loose stools; 4, diarrhea). The combined scores were then divided by three to obtain the final DAI. Colons were excised upon autopsy, and colon lengths were measured.

### BMDMs engraftment experiments

BMDMs were cultured as described earlier, and 5 × 10^5^ M0 or M2 polarized cells were injected intravenously into mice on day 0, 4, and 7 of DSS treatment.

### Cytokine assay

Cytokine levels (IL-1β, IL-6, and TNF-α) in sera of mice were measured by cytometric bead array immunoassay (CBA; BD Biosciences) according to the manufacturer’s instructions.

### Histology and immunohistochemistry

Colon tissue samples were fixed in PBS containing 10% neutral-buffered formalin. Paraffin-embedded sections (5 μm) were stained with hematoxylin and eosin. The degree of histological damage and inflammation was graded by a blinded veterinary pathologist. The following variables were scored: (i) amount of inflammation (0, none;1, mild; 2, moderate; 3, severe; 4, accumulation of inflammatory cells in the gut lumen), (ii) distribution of lesions (0, none; 1, focal; 2, multifocal; 3, nearly diffuse; 4, diffuse), and (iii) depth of inflammation and layers involved (0, none; 1, mucosa only; 2, mucosa and submucosa; 3, limited transmural involvement; 4, transmural). The overall histological score was the sum of the three variables (maximum score 12). Consecutive cryostat colon sections were used for immunohistochemistry to detect deposited Arg1 protein. The mean density was shown as an average integrated optical density/area using the Image-Pro Plus software (version 6) (Media Cybernetics, USA).

### Statistical analysis

All data are expressed as means ± SD. Comparisons between two groups was performed using the Student’s *t* test, whereas differences between >2 groups were performed using one-way ANOVA, (with *post hoc* comparisons using Dunnett tests) with GraphPad Prism version 4.0 statistical software. Differences were considered significant if *P* < 0.05.

## Additional Information

**How to cite this article**: Ji, J. *et al*. Microbial metabolite butyrate facilitates M2 macrophage polarization and function. *Sci. Rep.* **6**, 24838; doi: 10.1038/srep24838 (2016).

## Supplementary Material

Supplementary Information
