# Supplementary material for: Microbial metabolite butyrate facilitates M2 macrophage polarization and function
Source: Sci Rep. 2016 Apr 20;6:24838. doi: 10.1038/srep24838 (PMC4837405; doi:10.1038/srep24838)
Supplement: Supplementary Information [file srep24838-s1.pdf]

Microbial metabolite butyrate facilitates M2 macrophage polarization and function

Jian Ji[1,2,4], Dingming Shu[1,2,4], Mingzhu Zheng[3], Jie Wang[1,2], Chenglong Luo[1,2], Yan Wang[1,2], Fuyou Guo[1,2], Xian Zou[1,2], Xiaohui Lv[1,2], Ying Li[1,2], Tianfei Liu[1,2] & Hao Qu[1,2]*

[1]Institute of Animal Science, Guangdong Academy of Agricultural Sciences, 1 Dafeng 1st Street, Wushan, Tianhe District, Guangzhou 510640, China

[2]State Key Laboratory of Livestock and Poultry Breeding, Guangzhou 510640, China

[3]Institute of Immunology, Zhejiang University School of Medicine, Hangzhou, China

[4]Co-first author

* Correspondence: qhw03@163.com

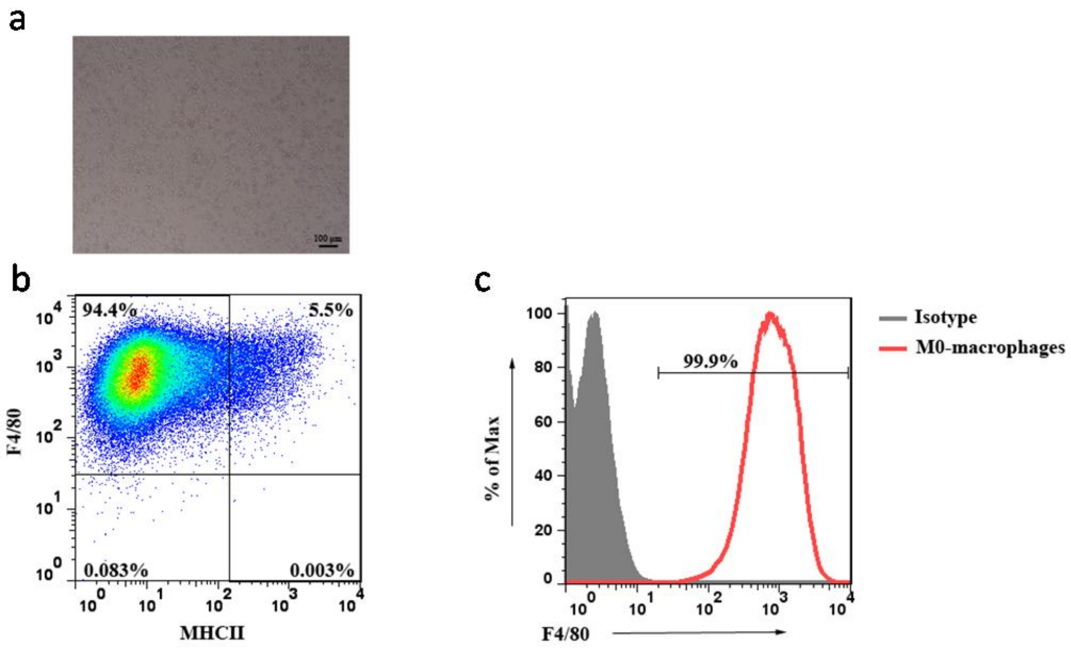

Supplementary Figure 1: Generation of bone marrow-derived macrophages. (**a**) Microscope image of 6 day-cultured BMDMs. (**b–c**) F4/80 surface expression was analyzed by flow cytometry.

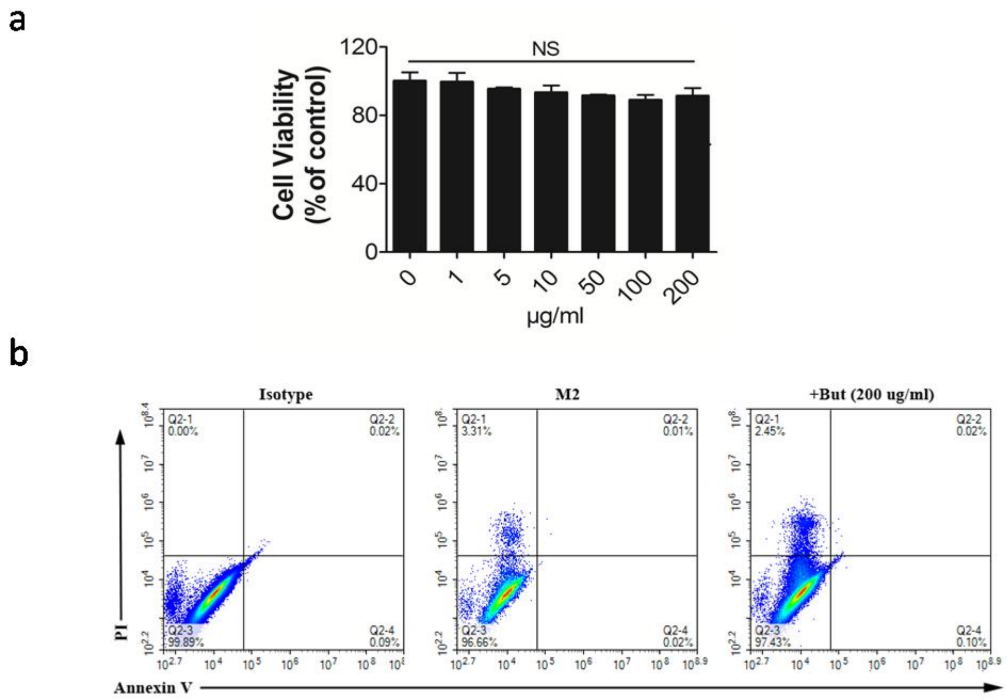

Supplementary Figure 2: The effect of butyrate treatment on the viability of M2-BMDMs. Differentiated M2-BMDMs were treated with butyrate for 24 h. Cellular viability was determined by the Cell Counting Kit-8 (CCK-8), and analysis of annexin V and propidium iodide (PI) staining by flow cytometry. (**a**) Cell viability was determined by the relative absorbance, untreated cells were used as controls. (**b**) Cells were double stained with annexin-V-FITC and PI and analyzed by flow cytometry. Data are shown as means ± SD for at least three independentexperiments.**P* <0.05, ***P* <0.01, ****P* <0.001 (*t* test).

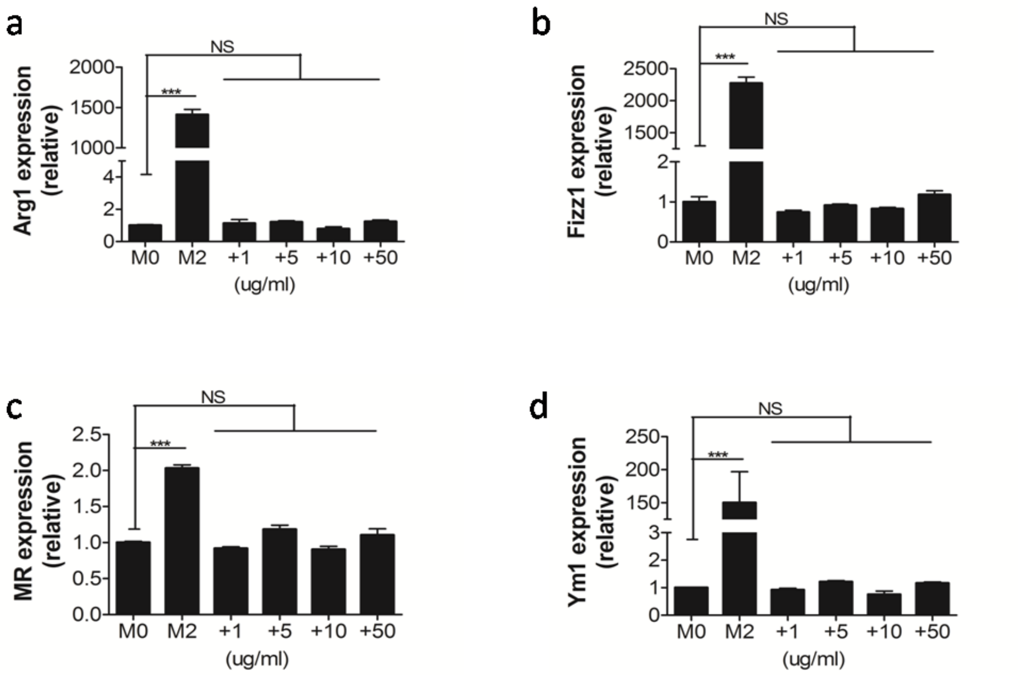

Supplementary Figure 3: Effect of butyrate on gene expression in M2-BMDMs. (**a–d**) Expression of *Arg1*, *Fizz1*, *Ym1*,and *MR* by M2-BMDMs after treatment with various concentrations of butyrate was measured by quantitative PCR. Data are shown as mean ± SD for at least three independent experiments.*P <0.05, **P <0.01, ***P <0.001 (t test).

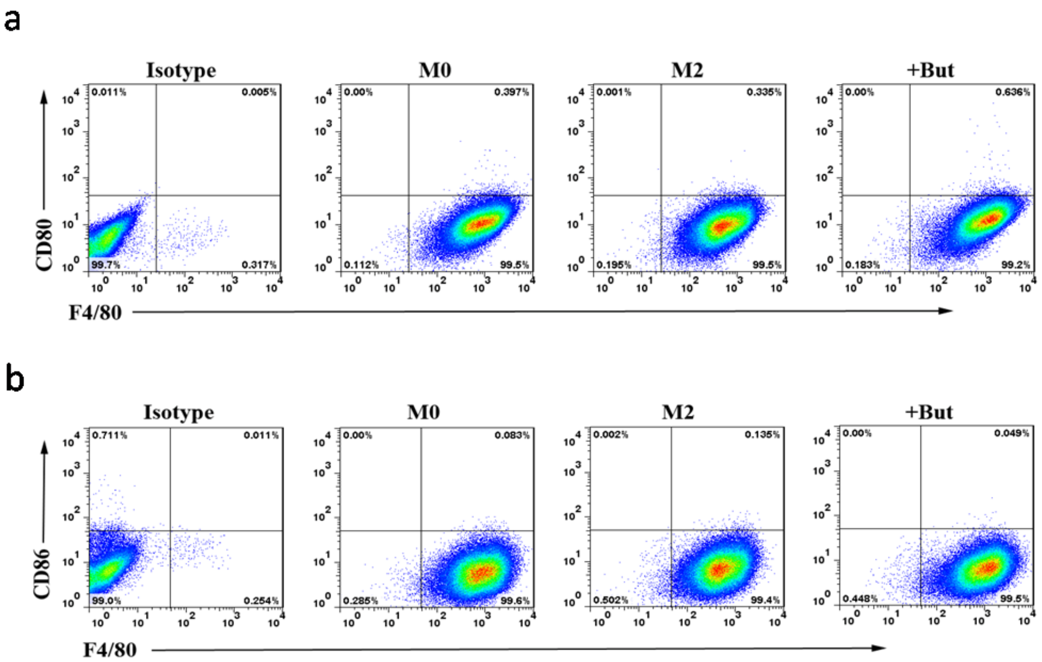

Supplementary Figure 4: The activation profiles of M2-BMDMs treated with butyrate. (**a–c**) M2-BMDMs were treated with butyrate for 12 h. Cells were harvested, washed, and the expression of CD80 and CD86 was analyzed by flow cytometry. Results are representative of three independent experiments.
